# Traumatic injury and perceived injustice: Fault attributions matter in a “no-fault” compensation state

**DOI:** 10.1371/journal.pone.0178894

**Published:** 2017-06-05

**Authors:** Liane J. Ioannou, Peter A. Cameron, Stephen J. Gibson, Belinda J. Gabbe, Jennie Ponsford, Paul A. Jennings, Carolyn A. Arnold, Stella M. Gwini, Nellie Georgiou-Karistianis, Melita J. Giummarra

**Affiliations:** 1 School of Psychological Sciences and Monash Institute of Cognitive and Clinical Neurosciences, Monash University, Faculty of Medicine, Nursing and Health Sciences, Clayton, Victoria, Australia; 2 Department of Epidemiology and Preventive Medicine, School of Public Health and Preventive Medicine, Monash University, Melbourne, Victoria, Australia; 3 Caulfield Pain Management and Research Centre, Caulfield Hospital, Caulfield, Victoria, Australia; 4 National Ageing Research Institute, University of Melbourne, Parkville, Victoria, Australia; 5 Farr Institute @CIPHER, Swansea University Medical School, Swansea, Wales, United Kingdom; 6 Monash-Epworth Rehabilitation Research Centre, Epworth Hospital, Richmond, Victoria, Australia; 7 Department of Community Emergency Health and Paramedic Practice, Monash University, Frankston, Victoria, Australia; 8 College of Health and Biomedicine, Victoria University, Footscray, Victoria, Australia; 9 Academic Board of Anaesthesia & Perioperative Medicine, School of Medicine Nursing & Health Sciences, Monash University, Clayton, Victoria, Australia; 10 Institute for Safety, Compensation and Recovery Research, Monash University, Melbourne, Victoria, Australia; University of Toronto, CANADA

## Abstract

**Background:**

Traumatic injury can lead to loss, suffering and feelings of injustice. Previous research has shown that perceived injustice is associated with poorer physical and mental wellbeing in persons with chronic pain. This study aimed to identify the relative association between injury, compensation and pain-related characteristics and perceived injustice 12-months after traumatic injury.

**Methods:**

433 participants were recruited from the Victorian Orthopedic Trauma Outcomes Registry and Victorian State Trauma Registry, and completed questionnaires at 12–14 months after injury as part of an observational cohort study. Using hierarchical linear regression we examined the relationships between baseline demographics (sex, age, education, comorbidities), injury (injury severity, hospital length of stay), compensation (compensation status, fault, lawyer involvement), and health outcomes (SF-12) and perceived injustice. We then examined how much additional variance in perceived injustice was related to worse pain severity, interference, self-efficacy, catastrophizing, kinesiophobia or disability.

**Results:**

Only a small portion of variance in perceived injustice was related to baseline demographics (especially education level), and injury severity. Attribution of fault to another, consulting a lawyer, health-related quality of life, disability and the severity of pain-related cognitions explained the majority of variance in perceived injustice. While univariate analyses showed that compensable injury led to higher perceptions of injustice, this did not remain significant when adjusting for all other factors, including fault attribution and consulting a lawyer.

**Conclusions:**

In addition to the “justice” aspects of traumatic injury, the health impacts of injury, emotional distress related to pain (catastrophizing), and the perceived impact of pain on activity (pain self-efficacy), had stronger associations with perceptions of injustice than either injury or pain severity. To attenuate the likelihood of poor recovery from injury, clinical interventions that support restoration of health-related quality of life, and adjustment to the impacts of trauma are needed.

## Introduction

Many injured persons face significant loss and suffering following traumatic injury, including functional impairment, financial insecurity, psychological distress, and disabling pain [1]. Perceiving that the injury event and outcomes were unfair may ultimately impact on the capacity to cope with and accept the consequences of the injury [2]. This is especially the case if the injury occurred as a consequence of reckless, negligent or intentional actions of another [3], or simply if the injured person believes that it was not their own fault. Perceptions of injustice have been shown to be a salient outcome in persons with persistent consequences of injury (e.g., patients seeking treatment for persistent pain) [4–6]; for an overview, see Sullivan, Scott [7]. Altogether, in persons who are experiencing persistent pain or disability after injury, perceived injustice is associated with more severe pain [8], maladaptive pain beliefs and difficulty coping with pain (e.g., catastrophizing) [9], poorer psychological functioning [4, 5, 10], worse disability [11], increased likelihood of engaging in litigation [6, 12], and failure to return to work [6]. While perceived injustice is strongly associated with worse outcomes, few studies have prospectively examined the factors leading to perceived injustice per se. This is an important question in its own right given that perceived injustice may have a significant impact on future behavior and wellbeing (e.g., efforts to return to work, seeking damages, suicidal ideation), and we need to identify ways in which we can improve individual experiences through trauma and health systems to attenuate injustice experiences.

The most consistent “injustice” characteristics examined in trauma outcome studies pertain to fault attribution, seeking legal representation, or the lodgment of a compensation claim to seek income replacement support, health care and/or lump sum damages. Attributing fault to someone, or something (e.g., an animal or environmental feature), other than oneself is frequently associated with poorer coping with illness [13], and worse health and work outcomes following injury [14, 15]. While external locus of causality may impact directly on coping with injury and illness, a range of other individual and contextual factors are likely to contribute to injustice experiences.

In particular, injured persons who are more likely to experience injustice are likely to possess a range of vulnerabilities that compound the impact of the injury. These include predisposing characteristics (e.g., fewer financial resources, lower education, less flexible work options, histrionic personality, trait anger), enabling characteristics (e.g., endorsement of injustice beliefs by friends, family, healthcare providers, employers or lawyers, compounding injustice experiences over time, e.g., from compensation system exposure) and characteristics directly indicative of perceived or objective injustice severity and the “need” for justice (e.g., severe injury, persistent pain, disability or psychological distress, perceived entitlement). In one of the few prospective studies to examine perceived injustice after injury, a range of predisposing (i.e., older age, lower education, lower income) and “injustice severity” characteristics (i.e., longer hospital stay and sustaining injury in violent crime or from penetrating trauma) were associated with higher injustice beliefs 12-months after admission to hospital for traumatic injury [16]. However, this former study, conducted in Texas, USA, did not measure or describe the type or access to compensation in this setting.

Compensable injury is frequently found to lead to worse psychological [17] and pain [18] outcomes, compared with non-compensable injury, and these outcomes are even worse for those who were not at fault for their injury [19] and in those who seek legal representation [20]. In many settings, entitlement to compensation is limited to claimants who can prove that another was at fault for their injury. In the present setting, however, in the state of Victoria, Australia, both the transport and workplace compensation systems are fundamentally no-fault systems. That is, all injured persons are entitled to health care, income replacement and compensation regardless of their role in the incident. Claimants who have sustained permanent impairment may also be entitled to lump sum compensation payments and, depending on the severity of their injury life-long income support, and if another was at fault, the claimant may also lodge for common law damages [21]. A recent cross-sectional study compared justice experiences in compensation claimants in the Victorian no-fault system, with those in the fault-based system in New South Wales, and found that fairness perceptions were higher in the no-fault system. Moreover, fairness perceptions were inversely associated with self-reported health-related quality of life [22]. Negative compensation-related experiences and stresses have been associated with worse pain and disability [23, 24]. Processes involved in eligibility for, and claiming compensation, such as paperwork, medicolegal assessments and approval of services, have secondary impacts on coping with the consequences of injury.

The present study aimed to identify the factors associated with perceived injustice at 12-months post-injury in persons admitted to hospital following traumatic injury in Victoria, Australia. This was an observational cohort study with a cross sectional design. Ultimately we aimed to determine whether characteristics at the time of injury, or hospital discharge (e.g., injury severity, fault, compensation status), could identify which patients would have higher perceptions of injustice, or whether these beliefs emerged alongside the persistence and difficulty coping with pain; disability and not returning to work; and mental health symptoms over time. We hypothesized that, in addition to attributing fault to another, entitlement to compensation, and having a more severe injury, outcomes relating pain severity, disability and difficulty coping with pain would be significant predictors of perceived injustice.

## Method

### Participant recruitment

Participants were recruited from the Victorian State Trauma Registry (VSTR)[25] and the Victorian Orthopedic Trauma Outcomes Registry (VOTOR)[26]. Participants were recruited if they attended The Alfred Hospital, a major trauma service hospital in Victoria and met the inclusion criteria for the trauma registries. The VSTR monitors major trauma cases and systems in Victoria, Australia, and collects data pertaining to pre-injury demographics, trauma and admission on all patients admitted to 138 hospitals in the state, and patient outcomes (6- and 12-months post injury) are collected through telephone interview.

The principle criteria for inclusion in VSTR are (a) admission to intensive care unit for >24 hours and mechanistically ventilated; (b) significant injury to two or more body regions (i.e., an abbreviated Injury Score (AIS) of >2 in two or more body regions) or a total Injury Severity Score (ISS) greater than 12; (c) urgent surgery within 24 hours for intracranial, intrathoracic or intra-abdominal injury, or fixation of pelvic or spinal fractures; (d) electrical injuries, drowning and asphyxia.

Patients are included in VOTOR if they have sustained an orthopedic (bone or soft tissue) injury and were admitted to one of four Victorian hospitals for > 24 hours. Patients who have soft tissue injuries that were managed conservatively do not enter VOTOR, and therefore were not eligible for participation in the present study. These recruitment sources ensured that the cohort were drawn from one of the two major trauma services in the state of Victoria, Australia, and details about the initial trauma or hospitalization were not reliant on patient recall.

Injured persons, admitted to The Alfred Hospital following injury in October 2012-October 2014, were invited to participate in the study by the registry interviewers, who were not part of this project team, at the conclusion of their 12-month registry telephone interview. Patients who required a proxy (e.g., due to brain injury) were not invited to participate, and patients with identified issues or distress were flagged by the trauma registry staff and the reason for distress was provided (e.g., death associated with injury, severe injury/disability). Only English-speaking participants aged 18–70 were eligible. Exclusion criteria were cognitive impairment, assessed qualitatively during trauma registry interview, or need for proxy. The study was approved by Alfred Hospital (study: 290/13) and Monash University (study: CF13/3276–2013001633) human research ethics committees, and participants provided written informed consent. For a full summary of the study procedures and materials, see doi: 10.17504/protocols.io.hqrb5v6.

### Procedures

#### Data linkage

VSTR and VOTOR trauma registries contain data pertaining to pre-injury demographics, trauma and admission data, and outcomes are assessed through telephone interview, which have been described in detail elsewhere [18, 26–28]. Registry data were collected at discharge from hospital and in interviews 6- and 12-months following injury. Participant data were extracted from the trauma registries, including patient demographics and injury information (i.e., trauma circumstances, injury coding and severity scoring, discharge location, compensation status) and some discharge outcomes at 12-months post-injury.

### Measures

Following the 12-month registry interview, we administered additional questionnaires either by telephone, online or in hardcopy to more comprehensively measure pain and mental health outcomes, perceived injustice, and compensation experiences.

#### Demographics

Participant demographic data included sex, age at injury/accident, education level, annual household income (12-months after injury) and comorbidity prior to injury. A neighborhood measure of socio-economic status (SES) was calculated from the residential postcode of potential participants using the Index of Relative Socio-Economic Advantage and Disadvantage (IRSAD) [29]. The IRSAD score is calculated based on typical education, employment and family structure in that area based on national census data. Each area is ranked nationally, with lower scores representing greater disadvantage.

#### Cause and severity of injury

Information about trauma included cause of injury, length of hospital stay (in days), admission to the intensive care unit (ICU), whether the injury was compensable, and work-status pre-injury and 12-months post-injury. Participants reported whether they attributed fault for the accident to themselves or another person (i.e. external). Participants also disclosed whether they had consulted a lawyer (whether or not the patient proceeded to litigation within 12-months of injury).

*Injury Severity Score (ISS)* was generated using the Abbreviated Injury Scoring (AIS) system [30]. For all patients, AIS was coded retrospectively by a trained and experienced AIS coder either employed by the health service trauma registry or the Victorian State Trauma Registry. The method of AIS coding is consistent across all health services, with coding occurring after definitive care admission to ensure that all information about the injury was available for accurate coding. The AIS coders were all trained in the rules and guidelines for AIS coding, including the ranking of sources and reliability of injury information. As AIS is not included in the VOTOR registry, AIS scores for 90 cases who were only registered to VOTOR and had sustained isolated limb injuries with an ISS <12, were assigned AIS codes based on the International Classification of Diseases (10) Australian Modification (ICD-10-AM) diagnosis codes. These cases were included to give a spectrum of relatively minor and major injuries. The methods followed in this study were in line with best practice in trauma and registry sciences, and valid for coding isolated limb injuries where the nature, location and type of injury is clear in the ICD-10 diagnosis codes and injury descriptions. The maximum AIS severity score was used in the present study to reflect the most severe injury, which ranged from 1 = ‘*minor’*, 2 = ‘*moderate’*, 3 = ‘*serious’*, 4 = ‘*severe’*, 5 = ‘*critical’* and 6 = ‘*unsurvivable*’.

Generally only one measure of injury severity is used due to the high correlation between length of hospital stay, ICU admission and ISS. However, we have used all three as patients in VSTR tend to have more severe multi-trauma injuries than VOTOR patients, and length of hospital stay only pertains to the first admission for definitive care, and in rare circumstances patients may be administratively re-admitted when they transferred to another ward (e.g., for management of infections or complications), however usually the total length of stay reflects the whole inpatient stay.

#### Perceived injustice

Perceived injustice was measured using the *Injustice Experience Questionnaire (IEQ)*, a 12-item scale measuring frequency of experiencing each of 12 feelings related to the injury and subsequent situation [1]. Items were rated on a 5-point Likert scale from 0 ‘*not at all’* to 4 ‘*all the time’*. The scale has subscales comprising Severity/Irreparability of Loss (6-items; Cronbach α = .90 in the present sample) and Blame/Unfairness (6-items; Cronbach α = .91). The total score reflects global perceptions of injustice, with scores ≥20 indicating clinical elevation [6].

#### Pain

Injustice experience was assessed using six pain outcomes: pain intensity, pain interference, pain-related disability, catastrophizing about pain, kinesiophobia and pain self-efficacy. The *Brief Pain Inventory (BPI)* comprises 11-point numerical rating scales of pain intensity (right now, least, worst, average) and interference with various aspects of daily life in the previous week [31]. Four items reflecting pain intensity were rated from 0 ‘*no pain*’ to 10 ‘*pain as bad as you can imagine*’, and seven items referring to pain interference were rated from 0 ‘*did not interfere’* to 10 ‘*interfered completely’*. A total score for each subscale was obtained by calculating the average of all item responses for each subscale (Cronbach α = .92 for pain severity and .95 for pain interference in the present cohort).

Pain-related disability was measured using the *Roland-Morris Disability Questionnaire (RMDQ)*, an 18-item scale requiring respondents to indicate whether certain actions/behaviors are difficult for them to undertake. It was originally developed for back-pain related disability; however, the present version reflected pain in general. The scale has high construct and content validity, internal consistency (Cronbach α: .92 in the present data) and test-retest reliability. A total score for this scale was calculated by counting the number of items to which the respondent had selected.

The *Pain Catastrophizing Scale (PCS)* was used to measure the tendency to have an exaggerated negative mindset in response to actual or anticipated painful experiences [32]. It is a 13-item scale that requests respondents to rate the degree to which they have certain types of thoughts and feelings when they are in pain. The 13 items are rated on a 5-point Likert scale from 0 ‘*not at all*’ to 4 ‘*all the time*’, with subscales of rumination, magnification and helplessness. For this study only a total score was used (Cronbach α = .95 in the present sample), which is calculated by adding all 13 item responses. Scores > 30 indicative of clinically elevated catastrophizing.

The *Tampa Scale of Kinesiophobia (TSK)* is a 17-item scale that measures fear of physical movement and activity that might result in painful injury and/or re-injury [33]. Each item is rated on a 4-point Likert scale from 1 ‘*strongly disagree*’ to 4 ‘*strongly agree*’ and a total score for the scale was calculated by adding the responses to all 17 items after inverting items 4, 8, 12 and 16 (Cronbach α = .84 in the present data). Scores > 37 are indicative of clinically elevated kinesiophobia.

The *Pain Self Efficacy Questionnaire (PSEQ)* comprises 10 items capturing one’s confidence in performing regular activities, including household chores, socializing and work, despite pain. This scale has high internal consistency (Cronbach α = .96 in the present data) and good test-retest reliability [34]. The items were answered on a 7-point Likert scale from 0 ‘*not at all confident*’ to 6 ‘*completely confident’*. A total score was calculated by adding the item scores, with higher scores indicating better self-efficacy. Scores < 20 are indicative of poor self-efficacy.

#### Health

Functional health and well-being was measured using the *Short Form Health Survey (SF-12)*, a validated and reliable 12-item scale that assesses respondent’s views about their health. The scale consists of two subscales reflecting physical health (physical component summary; PCS) and mental health (mental component summary; MCS). The PCS and MCS health scores range from 0 to 100, with 0 indicating the lowest level of health and 100 the highest.

### Statistical analyses

The data were analyzed with SPSS Version 22 (IBM Corp., Released 2013, Armonk New York) and tests were two-sided with α = .05. No single questionnaire item was missing more than 5% of responses, and the total amount of missing values in the data was also below 5%; therefore, missing data was not a significant concern [35].

Sub-scales of perceived injustice (outcome variable) were summarized using medians (with their lower and upper quartiles), because they were heavily skewed, and the median test was used to compare the equality of the group medians and differences in medians across sub-groups. The strength of the linear relationship between perceived injustice (total score), pain outcomes, the SF12 PCS and MCS were assessed using Pearson correlations prior to linear regression.

Predictors of perceived injustice were examined using hierarchical linear regression. The sample size was adequate for linear regression, which requires a minimum of 15 cases per predictor [36] and cases with missing data were excluded in a listwise manner. Furthermore, the types of factors included tend to have a fairly strong relationship with IEQ scores [1], and the sample size was more than sufficient to detect these modest relationships. Although the outcome variable was not normally distributed, the error variances were normally distributed and goodness-of-fit tests indicated that linear regression assumptions were satisfied. Hierarchical regression was used to establish the relationship between perceived injustice (outcome variable) and hierarchical steps comprising general subject characteristics (sex; age; education; work status prior to injury; comorbidities at baseline), injury severity (number of regions with moderate-critical injury; length of hospital stay), trauma (external fault attribution, lawyer engagement and compensation status) and function (mental and physical). The pain outcomes showed high multicollinearity, and so these variables were entered as a final step in six separate models to determine their unique contribution to perceived injustice while controlling for all other factors. The a-priori level of significance was p<0.05.

## Results

Between October 2013 and December 2014 a total of 732 individuals expressed interest in participating, of which 433 gave informed consent. We were unable to contact 70 potential participants, and 12 were ineligible giving a response rate of 65.4%. Name and contact details (phone number and address) were the only identifying information we had for patients who declined to participate. There was no difference in neighborhood SES (IRSAD Decile) between those who consented to participate (*Mdn* = 8, *IQR* = 4) and those who did not (*Mdn* = 7, *IQR* = 4) (*U* = 59,117.5, *p* = 0.09).

### Cohort overview

The participants were predominately male (74.8%), average age at time of injury was 44.8 years (*SD* = 14.2) and the majority had completed beyond secondary education (63.9%). The average time from injury to follow-up was 13.5 months (*SD* = 1.6) and at follow-up almost two thirds of the participants had a household income greater than AUD $60,000 per annum (60.9%). See Table 1 for participant characteristics. Most participants were working or studying in some capacity following injury (n = 302, 69.8%).

**Table 1:** Pre-injury participant characteristics and sub-group comparisons of perceived injustice scores.

			*Blame/unfairness sub-scale*		*Severity/irreparability of loss sub-scale*	
		*N (%)*	*Median (Q1; Q3)*	*p*	*Median (Q1*, *Q3)*	*p*
**Overall**		433	5 (0; 12)		8.5 (3; 15)	
**Sex** (n = 433)	Male	324 (74.8)	6 (0; 12)		9 (2; 15)	
	Female	109 (25.2)	4 (1;13)	0.490	7 (3;14)	0.268
**Age at injury** (n = 433)	<25 years	52 (12.0)	5.5 (1; 15)		9 (3; 17.5)	
	25–34 years	65 (15.0)	3 (0; 9)		6 (2; 12)	
	35–44 years	82 (18.9)	6 (2; 13)		10 (5; 16)	
	45–54 years	95 (21.9)	7 (1; 12)		8 (2; 13)	
	55–64 years	114 (26.3)	4.5 (0; 12)		10 (0; 13)	
	65+ years	25 (5.8)	4 (0; 10.5)	0.175	8.5 (3; 15)	0.187
**Education** (n = 432)	University degree	136 (31.5)	4 (0; 8)		6 (2; 13)	
	Diploma	140 (32.4)	7 (2; 13)		10.5 (4; 14.5)	
	Year 12	70 (16.2)	3 (0; 10)		7.5 (2; 13)	
	Years 9–11	86 (19.9)	8 (1; 15)	0.004	12 (4; 17)	<0.001
**Household income (12-months)** (n = 415)	$100,000+	135 (32.5)	3 (0; 8)		6 (2; 11)	
	$81–100,000	51 (12.3)	6 (1; 13)		8 (1; 13)	
	$61–80,000	67 (16.1)	5 (1; 12)		9 (3; 13)	
	$41–60,000	64 (15.4)	6 (1; 14.5)		9 (3; 15.5)	
	$20–40,000	98 (23.6)	7 (2; 15)	0.002	13 (4; 18)	0.004
**Comorbidity** (n = 433)	None	274 (63.3)	5 (0; 12)		8 (2; 15)	
	≥ one comorbidity	159 (36.7)	6 (0; 11)	0.392	9 (3; 15)	0.449

The cohort comprised 168 (38.8%) individuals who were injured in compensable incidents. The majority of participants had transport-related injuries (*n* = 173, 40.0%), injuries at home (*n* = 77, 17.8%), work (*n* = 45, 10.4%), as a victim of crime (*n* = 10, 2.3%) or other types of injuries (e.g., arising recreation such as sport or horse riding; *n* = 128, 29.6%). Less than a quarter of participants were admitted to ICU for their injury (*n* = 95, 21.9%) and two thirds (*n* = 292, 67.6%) were hospitalized for less than a week. Half of the participants admitted that they were at fault in the incident in which they were injured (*n* = 215, 50.1%). A fifth had consulted a lawyer (*n* = 88, 20.6%), of whom 74 (85.1%) believed they were not at fault for their injury.

## Injustice experience

The average injustice experience questionnaire score was 16.24 (SD = 13.64; median = 14.00, IQR: 4.00–27), and 159 (36.7%) participants had clinically elevated perceptions of injustice. Given the skew in the distribution of injustice scores, the median (IQR) subscale scores are presented in Tables 1 and 2. Median scores of perceived blame/unfairness scores and perception of severity/irreparability of loss decreased with higher levels of education and income. There were no significant differences in median subscale scores for age, sex or presence of comorbidities at injury.

**Table 2:** Perceived injustice sub-scales by injury and compensation-related outcomes.

			*Blame/unfairness sub-scale*		*Severity/irreparability of loss sub-scale*	
		N (%)	*Median (Q1*, *Q3)*	*p*	*Median (Q1*, *Q3)*	*p*
**Injury Severity** (max AIS score) (n = 433)	1–2	188 (43.4)	3 (0; 10.5)	0.002	7.5 (2; 14)	0.390
	3	171 (39.5)	6 (1; 12)		8.5 (2; 15)	
	4–5	74 (17.1)	7 (2; 15)		11 (5; 17)	
**Length of Hospital Stay** (n = 432)	1–2 days	158 (36.6)	3 (0; 9)	0.002	5 (1; 12)	<0.001
	3–6 days	134 (31.0)	6 (1; 12)		10 (3; 16)	
	7–13 days	82 (19.0)	6 (1; 12)		9 (4; 14)	
	14+ days	58 (13.4)	10 (4; 15)		13 (8; 18)	
**Admitted to ICU** (n = 433)	None	338 (78.1)	4 (0; 11)	0.071	8 (3; 14)	0.163
	Yes	95 (21.9)	6 (1; 15)		11 (3; 17)	
**Compensation status**(n = 433)	None	265 (61.2)	3.5 (0; 10)	<0.001	7 (2; 13)	0.002
	Compensable	168 (38.8)	7 (3; 16)		11 (4; 18)	
**Perceived Fault** (n = 429)	Self	215 (50.1)	2 (0; 7)	<0.001	6 (1; 13)	<0.001
	Not at Fault	214 (49.9)	8 (2; 16)		11 (4; 17)	
**Consulted a lawyer** (n = 428)	No	340 (79.4)	3.5 (0; 10)	<0.001	6 (2; 13)	<0.001
	Yes	88 (20.6)	15.5 (7; 21)		17 (11; 20)	
**Work status at 12-months** (n = 433)	RTW	302 (69.8)	4 (0; 10)	<0.001	7 (2; 13)	<0.001
	No RTW	73 (16.9)	13 (5; 20)		15 (9; 19)	
	Not working before injury	58 (13.4)	6 (1;13)		11 (3; 17)	

*Abbreviations*: AIS = Abbreviated Injury Severity; RTW = Return to work.

Higher perceptions of blame/unfairness and severity/irreparability of loss were reported by participants who were hospitalized for longer; received compensation; perceived that they were not at fault for the incident causing their injury; had consulted a lawyer; and were not working 12-months after injury; see Table 2. Surprisingly, perceptions of blame/unfairness, but not severity/irreparability, were higher in participants with higher injury severity scores.

## Predictors and covariates of perceived injustice

Perceived injustice was correlated with each pain and psychological outcome; see Table 3. That is, perceived injustice was associated with higher injury severity, pain severity, interference, catastrophizing and kinesiophobia, higher pain-related disability and poorer pain self- efficacy, and physical and mental functioning.

**Table 3:** Means, standard deviations and correlations between perceived injustice and pain and disability outcomes.

	*N*	*Mean* (*SD*)	Range of possible scores	Correlation between outcomes								
				1.	2.	3.	4.	5.	6.	7.	8.	9.
1. Perceived Injustice (Total)	431	16.26 (13.79)	0–48	1.00								
2. Pain Severity (BPI)	433	2.60 (2.05)	0–9	0.56**	1.00							
3. Pain Interference (BPI)	433	2.70 (2.54)	0–9	0.65**	0.82**	1.00						
4. Pain Catastrophizing (PCS)	432	9.70 (11.03)	0–42	0.66**	0.69**	0.74**	1.00					
5. Pain Self-Efficacy (PSEQ)	425	45.46 (14.23)	0–60	-0.65**	-0.65**	-0.70**	-0.63**	1.00				
6. Physical Function (SF12)	431	44.87 (11.82)	12–61	-0.56**	-0.62**	-0.71**	-0.56**	0.62**	1.00			
7. Mental Function (SF-12)	431	53.65 (10.44)	23–69	-0.39**	-0.36**	-0.39**	-0.47**	0.37**	0.21**	1.00		
8. Disability (RMDQ)	432	5.86 (5.42)	0–18	0.63**	0.69**	0.75**	0.64**	-0.70**	-0.71**	-0.29**	1.00	
9. Tampa Scale of Kinesiophobia (TSK)	431	37.50 (7.84)	17–68	0.55**	0.54**	0.56**	0.65**	-0.50**	-0.46**	-0.30**	0.57**	1.00

** Correlation is significantly different from 0 at α = 0.01 (2-tailed).

* Correlation is significantly different from 0 at α = 0.05 (2-tailed).

Hierarchical regression analyses significantly predicted the degree of perceived injustice, and explained a total of 52.2 to 57.3% of the variance in perceived injustice; see Table 4. Demographic “predisposing” factors accounted for only 6.2% of the variance in perceived injustice, and only education level was uniquely associated with perceived injustice. Injury characteristics explained an additional 4.6% of the variance, with both injury severity and length of hospital stay being significantly associated with perceived injustice. However, only length of hospital stay remained significant when adjusting for all demographic and other injury characteristics. Compensation- and justice-related factors explained a further 19.5% of the variance in perceived injustice. Fault and consulting a lawyer remained significant in the final model after adjusting for other covariates, however compensation status was no longer uniquely associated with perceived injustice in the fully adjusted model. Factors indicating the severity of the injury impacts on health-related quality of life, disability and pain outcomes explained a large portion of variance. Health-related quality of life (i.e., physical and mental component scores) at 12-months post-injury explained an additional 18.4% of the variance in perceived injustice. The association between perceived injustice and each pain-related outcome at 12-months post-injury was examined in separate models given the high multicollinearity between the pain outcomes. Pain catastrophizing explained the greatest amount of additional variance in perceived injustice (8.6%), followed by pain-related disability (7.1%) and pain self-efficacy (6.6%). Pain severity explained the least additional variance (3.5%) in perceived injustice.

**Table 4:** Hierarchical regression examining the effect of pain outcomes on perceived injustice (n = 413).

		Mean (sd)	β^*Unadj*^	β (95% CI)	Model statistics
**Step 1: Pre-injury characteristics**					R^2^ = 6.2%
**Sex**	Male (ref)	16.13 (13.32)	—	—	
	Females	16.55 (14.59)	0.42	-1.02 (-2.00, 4.05)	
**Age at injury**	<25 years (ref)	18.13 (15.38)	—	—	
	25–34 years	13.15 (12.45)	-4.98	-3.75 (-8.93, 1.43)	
	35–44 years	17.19 (13.52)	-0.95	-1.09 (-6.27, 4.08)	
	45–54 years	18.47 (13.45)	0.33	-0.29 (-5.28, 4.71)	
	55–64 years	15.03 (13.58)	-3.11	-4.09 (-8.99, 0.82)	
	65+ years	14.21 (13.01)	-3.93	-4.00 (-11.12, 3.12)	
**Education**	University degree (ref)	12.62 (12.13)	—	—	
	Diploma	18.02 (13.13)	5.40*	5.09 (1.88, 8.30)*	
	Year 12	14.89 (13.82)	2.27	1.54 (-2.39, 5.47)	
	Years 11 and below	20.08 (15.22)	7.46*	7.02 (2.94, 11.09)*	
**Work status before injury**	Unemployed (ref)	17.68 (14.27)	—	—	
	Working	16.02 (13.55)	-1.67	-1.36 (-5.60, 2.89)	
**Comorbidity**	None (ref)	15.99 (13.60)	—	—	
	≥ one comorbidity	16.67 (13.75)	0.68	0.17 (-2.62, 2.96)	
**Step 2: Injury outcomes**					ΔR^2^ = 4.6%; p<0.001
**Injury Severity** (count of regions with moderate-critical injuries)			2.01*	1.14 (-0.06, 2.33)	
**Length of Hospital Stay**			0.39*	0.29 (0.14, 0.44)*	
**Step 3: Compensation-related outcomes**					ΔR^2^ = 19.5%; p<0.001
**Compensation status**	None (ref)	13.66 (12.51)			
	TAC/WorkSafe	20.31 (14.39)	6.65*	0.49 (-2.30, 3.28)	
**Fault**	Self (ref)	11.94 (11.10)			
	Not at Fault	20.50 (14.62)	8.57*	4.33 (1.93, 6.74)*	
**Consulted a lawyer in first 12-months**	No (ref)	12.85 (11.70)			
	Yes	29.07 (13.14)	16.22*	13.00 (9.52, 16.47)*	
**Step 4**: Functional status					ΔR^2^ = 18.4%; p<0.001
SF-12 Physical functioning			-0.67*	-0.43 (-0.54, -0.33)*	
SF-12 Mental functioning			-0.56*	-0.38 (-0.50, -0.27)*	
**Step 5: Pain-related outcomes (entered into the separate model)**					
Pain severity (BPI)			3.72*	1.75 (1.07, 2.44)*	ΔR^2^ = 3.5%; p<0.001
Pain interference (BPI)			3.50*	2.05 (1.45, 2.66)*	ΔR^2^ = 6.0%; p<0.001
Pain Catastrophizing (PCS)			0.82*	0.50 (0.39, 0.61)*	ΔR^2^ = 8.6%; p<0.001
Tampa Scale of Kinesiophobia (TSK)			0.91*	0.48 (0.34, 0.62)*	ΔR^2^ = 6.2%; p<0.001
Pain Self-Efficacy (PSEQ)			-0.61*	-0.34 (-0.45, -0.22)*	ΔR^2^ = 6.6%; p<0.001
Disability (RMDQ)			1.59*	1.02 (0.75, 1.28)*	ΔR^2^ = 7.1%; p<0.001

*Abbreviations*: ref = reference category; SE = standard error; SF-12 = 12-item Short Form

^*^ H_0_: β = 0; p<0.05.

## Discussion

Perceptions of injustice may arise after traumatic injury, comprising beliefs that the injury and its consequences are unfair, severe, and irreparable or that another is to blame. While perceived injustice is recognized as playing a key role in long-term adaptation to the consequences of injury, few studies have examined which predisposing, enabling or justice-related characteristics are associated with the degree of injustice perceived. The present study found that potentially predisposing demographic characteristics have only a small association with attributions of blame or perceived irreparability, with higher injustice experiences associated with lower education. There were no significant associations between injustice beliefs and sex, age, or pre-injury health or work status before injury. Potential “enabling” and justice-related characteristics of the injury, and injury outcomes, played a much greater role in injustice beliefs. Specifically, those who had lower household income, and those who failed to return to work, 12-months post-injury had higher injustice beliefs, highlighting the key role of these economic “enabling” factors in the capacity to cope with the impacts of injury. Moreover, attribution of fault to another, consulting a lawyer, health-related quality of life, disability and the severity of pain-related cognitions explained the majority of variance in perceived injustice. Injury severity and pain severity had only small associations with injustice beliefs. Altogether, the present findings highlight that health-related quality of life after injury, as well as the level of emotional distress caused by pain, and perceived impact of pain on activity, have a much stronger association with perceptions of injustice than the severity of the injury or pain per se.

Just over a third of the present cohort had clinically elevated perceptions of injustice, and at the group level perceived injustice ratings were lower than those reported in studies where all patients have chronic pain conditions [2, 4–6, 37]. The differences likely lie in the fact that although most of the participants in this cohort had sustained very serious injuries; they were prospectively recruited 12-months after hospital admission, via well-established trauma registries, whereas many other studies into perceived injustice selectively recruit persons who were disabled, or were seeking treatment for pain. Nonetheless in the present sample, as in previous studies [1, 4, 6–8, 11, 16], perceived injustice was associated with worse pain catastrophizing, interference, kinesiophobia, self-efficacy, severity and pain-related disability. Ultimately, the association between perceived injustice and potential stable impacts 12-months post-injury on pain and function are likely to be bidirectional, and difficult to anticipate from patient demographic or injury characteristics at the time of hospital admission.

### Compensation, fault and perceived injustice

The present findings suggest that engaging with the compensation system did not have a unique association with global perceptions of injustice 12-months following injury. Rather, the experience of perceived injustice was more strongly associated with sustaining an injury that required a longer hospitalization, believing that another was at fault and consulting a lawyer. Injury severity was only associated with appraisals of blame/unfairness, but not perceptions of severity and irreparability of losses sustained. This suggests that the negative injury outcomes often found to be associated with external fault attributions may be compounded in those with more severe injuries. In the present study, therefore, it appears that although having a compensation claim in a no-fault system contributed to injustice beliefs it did not do so independently from other injury-, injustice-related factors.

Perceived injustice was especially elevated in those who had reported that another person was at fault, or consulted a lawyer. While we measured whether participants had sought legal advice and/or representation, other studies have found that the intention to litigate is associated with higher perceptions of injustice (e.g., after spinal cord injury [12]. Although the present setting has no-fault compensation systems for work and transport injury, and claimants do not need to prove another was at fault to be eligible, injured persons often nonetheless seek legal representation to assist with their transport or workplace injury claim. Moreover, while persons who have sustained a permanent impairment are entitled to lump sum payment for impairment, and lifetime income support, depending on the degree of impairment, regardless of their role in the injury incident if another was at fault, they may also seek common law damages via the compensation system for the impact of the injury. Outside of the context of compensable injury, injured persons may require legal representation to pursue common law claims for public liability or victims of crime cases.

Litigation and lawyer involvement in recovery from injury has previously been explored in several contexts, and it is consistently associated with worse pain outcomes, greater disability and poorer psychological functioning [38–40]. To our knowledge this is the first study to demonstrate that consulting a lawyer was associated with perceived injustice when controlling for attributions of fault and injury severity. Given that many injured persons may still have been in relatively early phases of their claim at 12-months post-injury, we speculate that those who had already consulted with a lawyer had a high need for justice. In some cases, however, consulting a lawyer can increase feelings of *in*justice (e.g., at least one participant said that the lawyer would not take on their case as their impairment was not severe enough), whereas other cases could have felt a greater sense of justice if the lawyer had already yielded benefits (e.g., lump sum payments for impairment). Moreover, it is possible that those consulting a lawyer reported greater disability or pain simply to enhance their likelihood of receiving compensation (e.g., on the advice of their solicitor), or because they were distressed and/or had an increased tendency to catastrophize.

### Clinical implications

While we did not find a unique association between compensation and perceived injustice, aspects of compensable injury, and engagement with the compensation system that were not examined in this study may play a role in perceptions of fairness and injustice [22, 41]. For instance, a recent study with psychologists highlighted that client perceptions of procedural injustice, especially undergoing independent medical examinations, may play a major role in psychological recovery of injured workers [42]. It is important to note that perceptions of injustice extend beyond attributing fault to another person, and include many additional experiences and interactions over time, with the most frequent sources of injustice being employers and colleagues, healthcare providers and insurers, friends and family, and society as a whole [43]. Understanding the factors impacting on experiences of justice, and injustice, should therefore be a high priority for health care providers and compensation systems. Specifically, screening for perceptions of blame and fault, and the severity and stability of the impacts of injury (e.g., risk of unemployment due to pain and disability) relative to available client resources (e.g., household income, education), may assist in identifying injured persons who are more likely to hold or develop perceptions of injustice.

Given that perceived injustice was associated with greater pain and pain-related disability, it is important to address these beliefs during treatment and rehabilitation after injury. There is some evidence that successful rehabilitation helps to attenuate injustice beliefs, however, it may also be necessary to directly address injustice beliefs during treatment. It is important to acknowledge client feelings of injustice, however, endorsing those beliefs may reinforce maladaptive behaviors [44]. Care must therefore be taken to focus on building resilience to continued sources of injustice, and to enhance the injured person’s sense of acceptance and responsibility over their recovery. Suitable treatments may include forgiveness-oriented [45], emotion regulation or anger management training, and methods that improve emotional awareness and regulation (e.g., mindfulness-based therapy) [46].

### Limitations

Several limitations of this study should be noted. It was a cross-sectional study and thus causal relationships cannot be determined. The cohort had slightly higher socio-economic status than the general population, with slightly higher post-school qualifications [47] and annual income than the national average [48]. The sample socioeconomic characteristics were also slightly higher than other trauma samples [14], which may limit the generalizability of the findings. Pain severity and disability were lower than levels typically found for persons seeking treatment for chronic pain (i.e., 5.7 versus 12.3 for pain disability; [49]) and the cohort may therefore not have been severely disabled due to pain despite a large proportion having sustained quite severe injuries.

Given that psychological and physical health outcomes are relatively better in a no-fault compensation system compared with fault-based systems (e.g., see [50, 51]), the present findings may not generalize to other jurisdictions with fault-based compensation systems. For instance, in fault-based compensation systems injured persons may experience additional factors that impact on injustice beliefs, such as having to prove that they were not at fault, being denied access to compensation, or having to undergo civil proceedings to receive a lump sum compensation payment to cover loss of earnings and/or health care needs. However, even in a fault-based system patients who attribute fault to another have worse recovery outcomes than those who were at fault [14,52]. Thus, although the present findings may be specific to the Victorian health and compensation setting, it appears that that perceptions of injustice are largely due to human factors rather than the system, per se.

## Conclusion

The present findings confirm and extend our understanding of perceived injustice following traumatic injury. Even when controlling for injustice aspects of injury (i.e. fault, lawyer involvement, compensable injury), difficulty coping with pain and greater psychological distress were strongly associated with elevated perceptions of injustice. In particular, we show that even in a system where all injured persons are entitled to compensable income replacement and healthcare, regardless of who was at fault, external attribution of fault is nonetheless associated with elevated perceptions of injustice. Worse health-related quality of life, and persistent disabling pain and pain-related cognitions (e.g., catastrophizing, kinesiophobia, and self-efficacy) were associated with higher perceptions of injustice, and are key characteristics that could be used as a focus of early interventions and to identify injured-persons at risk of a poor injury outcome.
